# Behavior of Four Olive Cultivars During Salt Stress

**DOI:** 10.3389/fpls.2019.00867

**Published:** 2019-07-05

**Authors:** Luca Regni, Alberto Marco Del Pino, Soraya Mousavi, Carlo Alberto Palmerini, Luciana Baldoni, Roberto Mariotti, Hanene Mairech, Tiziano Gardi, Roberto D’Amato, Primo Proietti

**Affiliations:** ^1^Department of Agricultural, Food and Environmental Sciences, University of Perugia, Perugia, Italy; ^2^Institute of Biosciences and Bioresources, National Research Council, Perugia, Italy

**Keywords:** salt stress, olive, photosynthesis, proline, antioxidant enzyme

## Abstract

Olive is considered as a moderately salt tolerant plant, however, tolerance to salt appears to be cultivar-dependent and genotypic responses have not been extensively investigated. In this work, saline stress was induced in four olive cultivars: Arbequina, Koroneiki, Royal de Cazorla and Fadak 86. The plants were grown in 2.5 l pots containing 60% peat and 40% of pumice mixture for 240 days and were irrigated three times a week with half-strength Hoagland solution containing 0, 100 and 200 mM NaCl. The effects of salt stress on growth, physiological and biochemical parameters were determined after 180, 210, and 240 days of treatment. Saline stress response was evaluated in leaves by measuring the activity of GSH and CAT enzymatic activity, as well as proline levels, gas exchanges, leaves relative water content and chlorophyll content, and proline content. All the studied cultivars showed a decrease in Net Photosynthesis, leaves chlorophyll content and plant growth (mainly leaves dry weight) and an increase in the activity of GSH and CAT. In addition, the reduction of proline content in leaf tissues, induced an alteration of osmotic regulation. Among the studied cultivars Royal and Koroneiki better counteracting the effects of saline stress thanks to a higher activity of two antioxidant enzymes.

## Introduction

Environmental conditions may strongly impact plant crop growth (Kachaou et al., 2010; Feller and Vaseva, 2014; Pandolfi et al., 2017). In particular, abiotic constraints, such as drought, soil salinity and extreme temperatures, which cause water depletion in cells, are responsible for a large proportion of losses in agricultural productivity (Bose et al., 2014).

In order to overcome water shortages and to satisfy the increasing water demand for agricultural development, the use of water of low quality (brackish, reclaimed, drainage) that frequently has an high salinity level is becoming important in many countries (Chartzoulakis, 2005).

In particular, plants under high salinity conditions are subject to significant physiological and biochemical changes, for example a marked decrease in photosynthesis rate and transport of salt ions from roots to shoots (Ben Ahmed et al., 2009; Anjum et al., 2011; Singh and Reddy, 2011; Goltsev et al., 2012; Abdallah et al., 2018). A major biochemical alteration, also induced by other types of stress, is the production of reactive oxygen species (ROS) (Gill and Tuteja, 2010; Boguszewska and Zagdańska, 2012; Ozgur et al., 2013; Bose et al., 2014). An excess of ROS leads to lipid peroxidation, inhibition of enzymes, and modifications of nucleic acids (Proietti et al., 2013; Bose et al., 2014; Tedeschini et al., 2015). Under stress conditions, plants can nonetheless develop tolerance, that is, the ability to adequately survive, and often prosper, under an unfavorable environment, following a robust production of antioxidant enzymes (Ben Ahmed et al., 2009; Bhaduri and Fulekar, 2012; Keunen et al., 2013). Among these enzyme, superoxide dismutase (SOD), ascorbate peroxidase (APX), and glutathione reductase (GSH) are localized in chloroplasts and mitochondria (Pang and Wang, 2010; Del Buono et al., 2011; Proietti et al., 2013), whereas catalase (CAT) and guaiacol peroxidase (GPX) are generally present within microbodies and cytosol, respectively (Bhaduri and Fulekar, 2012; Hameed et al., 2013; Nath et al., 2016).

Mechanistically, tolerance may also include osmotic adjustments at cellular level (Ayala-Astorga and Alcaraz-Meléndez, 2010). Some plants implement this process by increasing the amount of solutes and lowering the water potential of root cells, thereby counteracting the water outflow. These substances, reported as osmolytes, can accumulate in large amount, but do not generally interfere with enzymatic activities and cytoplasmic pH, due to their zwitterionic nature. Osmolytes commonly used by plants are sugars, alcohols, quaternary amines, betaine, glycine and proline (Warren, 2014). In this regard, the concentration of proline in leaves and roots was reported as a response by the olive tree to saline stress (Ayala-Astorga and Alcaraz-Meléndez, 2010; Hayat et al., 2012; Iqbal et al., 2014; Abdallah et al., 2018). In fact, proline facilitates water retention in the cytoplasm and, therefore, its concentration is indicative of response to saline stress (Ben Ahmed et al., 2009; Gupta and Huang, 2014). Cultivated olive (*Olea europaea* subsp. europaea var. europaea) is a long-living, evergreen, thermophilic species. In the Mediterranean basin where olive is mostly cultivated salinity is becoming a relevant problem due to high rates of evaporation and insufficient leaching (Mousavi et al., 2019). In addition in costal areas the need for water of good quality for urban use is increasing while there is a large amount of low quality water mostly saline (EC > 2.0 dS m^-1^) that can be use for irrigation (Chartzoulakis, 2005). Olive is considered as a moderately salt tolerant plant and the tolerance appear to be cultivar dependent (Rugini and Fedeli, 1990). The olive crop counts a very rich varietal heritage (Mousavi et al., 2017) but genotypic responses of olive to NaCl salinity have not been extensively investigated, and only few works have been published (Al-Absi et al., 2003; Chartzoulakis, 2005). In this context it’s important to select cultivars that may give good performance when cultivated in soil with salinity problems or irrigated with saline water. Among the cultivars studied in the present work Arbequina and Koroneiki cultivars are the subject of increasing interest given their adaptability to super-intensive cultivation systems (Proietti et al., 2015). The identification of saline-resistant cultivars is of particular interest, especially for those cultivation systems, such as the super-intensive, which require large quantities of water as the availability of non-saline water will decrease dramatically in the future due to climate change.

The purpose of this work was to study the behavior of different olive cultivars during saline stress by analyzing the activity of the GSH and CAT enzymes, the proline content and the plant growth parameters.

## Materials and Methods

### Plant Material, Growing Conditions and Salt Treatments

Sixty own-rooted plants for each olive cultivar Fadak 86, Royal de Cazorla (referred along the text as “Royal”), Koroneiki and Arbequina were used (20 plant replicates per treatment). Two-years old plants, approximately 1.3–1.5 m tall, were grown in greenhouse in black plastic pots (volume 2.5 L) containing a substrate composed of 60% peat and 40% pumice (w/w). Plants were irrigated three times a week, for 3 months, using half-strength Hoagland solution in the absence of salt. Subsequently, for the following 8 months from February, plants were irrigated three times a week with half-strength Hoagland solution containing 0, 100, and 200 mM NaCl, respectively. The salinity levels used were high since 137 mM NaCl has been is the tolerance limit for olive trees (Rugini and Fedeli, 1990) and were chosen accordingly to previous studies on salt stress in olive (Therios and Misopolinos, 1988; Tattini et al., 1995; Ben Ahmed et al., 2008). At beginning of treatment, to prevent osmotic shock, salt was added using daily increments of 25 mM up to the target levels. Electrical conductivity was determined weekly in the leaching solution with the conductometer “Hanna Instruments- HI 9033,” giving values of about 1.2, 12.4, and 21.4 dS m^-1^ in relation to the 0, 100, and 200 mM NaCl group, respectively, thus confirming that an irrigation rate with a leaching fraction of 20–30%, ensures a stable salinity level in pots throughout the course of the experiment (Perica et al., 2008).

Plants were exposed to natural light inside the greenhouse, and a ventilation system was automatically engaged by air temperature not exceeding 35°C.

During the entire course of the experiment, minimum and maximum daily temperatures ranged between 9.4 and 15.2°C, and 10.4–30.4°C, respectively.

### Plant Growth

At the beginning of the experiment and at 240 days after treatment (DAT), five plants for each treatment (including control plants) were removed from the substrate, roots were washed with deionized water and separated into different parts. For each plant, basal diameter, total height, number of lateral shoots, total length of lateral shoots, total leaf area and fresh and dry weight (FW and DW) of roots, lateral shoots (after removing the leaves) and stems (principal axis) were determined. DW was obtained by oven-drying at 95°C until constant weight was achieved.

At the end of the experiment the relative growth rate (RGR) was calculated as follows (Hoffmann and Poorter, 2002):

$$RGR=\frac{\ln\;(v_{2})-\ln\;(v_{1})}{t_{2}-t_{1}}$$

where: ln = natural logarithm; *v*_2_ e *v*_1_ = plant DW at the end (*t*_2_) and at the beginning (*t*_1_) of the experiment.

### Leaf Net Photosynthesis (Pn), Stomatal Conductance (gs), Leaf Transpiration Rate (E), and Sub-Stomatal CO_2_ Concentration (Ci)

Photosynthesis, gs, E and Ci were determined at 180, 210 and 240 DAT in 15 leaves for each combination (cultivar + treatment). Leaf gas exchange rates were measured using a portable IRGA (ADC-LCA-3, Analytical Development, Hoddesdon, United Kingdom) and a Parkinson-type assimilation leaf chamber. Leaves were enclosed in the leaf chamber and perpendicularly exposed to sun’s rays inside the greenhouse. PPFD was always higher than 1,200 μmol m^-2^ s^-1^ (within the 1,500–1,900 range), which is known to be over the saturation point in olive (Proietti and Famiani, 2002). The flow rate of air passing through the chamber was kept at 5 cm^3^ s^-1^. During gas-exchange measurements, the external CO_2_ concentration was about 385 cm^3^ m^-3^ and the air temperature inside the leaf chamber was 2–4°C higher than outside. Measurements were taken under steady state conditions (about 30 s). Leaves were then returned to the laboratory for area measurements using a Delta-T Image Analysis System (“Delta-T Devices,” Cambridge, United Kingdom) and Pn, gs and E were expressed in relation to the leaf area.

### Leaf Water Status and Chlorophyll Content

For each treatment, relative water content (RWC) was determined from leaves of five plants (three leaves each), collected at 180, 210, and 240 DAT. Leaves were detached, sealed in a plastic bag, and taken immediately to the laboratory to determine leaf water status according to the procedure previously described (Proietti et al., 2013).

Total chlorophyll content was determined by the portable SPAD-502 chlorophyll meter, which allows rapid, non-destructive measurements (Boussadia et al., 2011).

### Chemical Analysis

#### Glutathione Reductase (GSH)

The enzymatic activity was measured by a modification of the previously published method (Flohé and Günzler, 1984) using H_2_O_2_ as substrate. GSH activity was measured in olive leaves (five leaves of about 1 g total for each combination cultivar+treatment) homogenized in 5 ml of KNaHPO_4_ (0.1 M) buffer at pH 7.0 containing EDTA 1 mM, with an ultra-Turrax T25 homogenizer (Tanke and Kunkel Ika Labortechnik) for 3 min in ice. The supernatant obtained by centrifugation (10 min at 3,000 rpm) was used as the source of enzyme activity.

The reaction mixture consisted of 0.2 mL of the extract supernatant, 0.4 ml of GSH (0.1 mM) and 0.2 ml of KNaHPO_4_ (0.067 M) containing EDTA 1 mM. The reaction mixture was kept at 25°C for 5 min, after which the reaction was started by adding 0.2 ml of H_2_O_2_ (1.3 mM), and then stopped 10 min later with 1 ml of trichloroacetic acid.

The mixture was cooled in ice for 30 min and centrifuged at 3000 rpm for 10 min; the supernatant (0.48 ml) was placed in a cuvette containing 2.2 ml of 0.32 M Na_2_HPO_4_ and 0.32 ml of 1 mM DTNB (Sigma-Aldrich), and read after 5 min in a Beckman spectrophotometer set at 412 nM.

#### Catalase (CAT)

Catalase activity was carried out in olive leaves (five leaves for each combination cultivar+treatment) homogenized for 3 min in ice by an ultra-Turrax T25 in 5 ml of 0.2 M Tris buffer (pH 7.8) containing 0.13 mM EDTA and 80 mM PVP. The homogenates were centrifuged at 3,000 × *g* for 10 min, after which the supernatants were used to measure CAT activity. CAT activity was determined based upon the consumption of hydrogen peroxide (coefficient of extinction 39.4 M^-1^ cm^-1^) at 240 nM for 2 min (Kraus et al., 1995).

The reaction mixture contained 2 ml of a 100 mM NaH_2_PO_4_/Na_2_HPO_4_ buffer (pH 6.5), and 0.05 ml of the extract. The reaction was started by adding 0.01 ml of 30% (w/v) hydrogen peroxide.

#### Proline

The determination of proline in leaves (five leaves for each combination cultivar + treatment of about 1 g total) was performed by HPLC using a Jasco 880-PU instrument equipped with a Jasco 821-FP fluorometric detector. The HPLC procedure was carried out according to the method described (Palmerini et al., 1985). Proline was measured in leaves homogenized in 5 mL of ultra-pure H_2_O with an ultra-TurraxT25 for 3 min in ice.

The extract supernatant (1 ml) was deproteinized with 0.2 ml of HClO_4_ (20% v/v) in ice, centrifuged at 8000 rpm for 5 min, and finally neutralized with 0.2 ml of KOH (20% by weight).

The supernatant (0.05 ml) was mixed with 0.15 ml (0.4 M) of borate pH 9, 0.05 ml of o-phthalide chloride (OPA) (150 mM) in methanol and 0.1 ml of 7-chloro-4-nitrobenzo 2 ossa-1,3-diazolo (NBD-Cl) (25 mM) in methanol. The reaction, set at 60°C for 3 min, was stopped in ice with 0.1 ml HCl (1 M).

The derivatized sample (0.02 ml) was injected into a HPLC Lichrosor RP-18 column (15 cm × 4.6 mm ID) and eluted under isocratic conditions with H_2_O/CH_3_CN (93/7), used as the mobile phase. The solvents used were previously passed through a 0.22-micron filter (Millipore Corporation).

NBD-derivatives were determined at 470 nM (excitation) and 530 nM (emission). NBD-proline was eluted in 6.5 min and quantified using a standard proline solution. Proline (0.043 M) and hydroxy-proline (0.038 M) standards were diluted 1 to 100 in H_2_O, derivatized with NBD-Cl and analyzed by the same HPLC method to generate reference data.

### Statistical Analysis

All statistical analyses of data were performed using Graph Pad Prism 6.03 software for Windows (La Jolla, CA, United States). Tests for variance assumptions were conducted (homogeneity of variance by the Levene’s test, normal distribution by the D’Agostino-Pearson omnibus normality test). Significance of differences were analyzed by Fisher’s least significant differences test, after the analysis of variance according to the randomized complete factorial design. Differences with *p* < 0.05 were considered significant (Supplementary Tables S1–S14). The coefficient of variation (CV) was determined for each trait.

## Results

### Leaf Net Photosynthesis (Pn), Stomatal Conductance (gs), Leaf Transpiration Rate (E), and Sub-Stomatal CO_2_ Concentration (Ci)

Plants treated with 100 and 200 mM NaCl showed lower values of Pn compared to control, especially at 210 and 240 days after treatment start (DAT). The Pn decrease in stressed plants started from 180 DAT, and the most significant impact was observed in “Arbequina,” and “Fadak 86” treated withf Pn compared to control, 100 and 200 mM NaCl and the plants of the same cultivars treated with 200 mM NaCl died at 220 DAT. Ci increased at 100 and 200 mM NaCl and the higher values were observed in Arbequina and Fadak 86 gs and E decreased at 200 mM.

As a general trend, in stressed plant, the decrease in Pn was accompanied by a decrease in gs. On the other hand, gs decrease is related with an increase in Ci and a decrease in E (Table 1).

**Table 1:** Gas exchanges in four cultivars treated with 0, 100, and 200 mM NaCl.

	Pn μmol(CO_2_) m^-2^ s^-1^	E mmo l(H_2_O) m^-2^ s^-1^	gs mmol(H_2_O) m^-2^ s^-1^	Ci μmol mol^-1^
**180 DAT**				
Fadak 86-0	12.14 a	3.50 a	246.50 a	297.21 a
Fadak 86–100	11.67 a	3.55 a	236.38 a	284.46 a
Fadak 86–200	11.40 a	3.60 a	232.47 a	281.67 a
Royal-0	13.80 a	2.93 a	147.97 a	209.54 a
Royal-100	12.03 a	2.43 a	127.96 b	213.80 a
Royal-200	10.15 b	2.25 a	128.62 b	201.41 a
Koroneiki-0	14.10 a	3.85 a	252.65 a	260.69 b
Koroneiki-100	10.69 b	3.81 a	209.77 b	312.56 a
Koroneiki-200	9.31 b	3.51 a	180.34 b	359.19 a
Arbequina-0	13.05 a	3.38 a	184.47 a	254.94 a
Arbequina-100	11.63 b	2.32 b	174.70 b	244.43 a
Arbequina-200	11.01 b	2.33 b	170.79 b	251.64 a
**210 DAT**				
Fadak 86-0	11.75 a	2.12 a	162.53 a	288.21 b
Fadak 86–100	7.20 b	1.38 b	72.04 b	346.47 a
Fadak 86–200	5.47 b	1.35 b	68.78 b	332.03 a
Royal-0	6.72 a	0.69 a	21.60 a	112.07 a
Royal-100	6.02 a	0.72 a	27.06 a	101.43 a
Royal-200	5.17 b	0.70 a	22.18 a	117.32 a
Koroneiki-0	9.30 a	2.12 a	90.75 a	241.55 b
Koroneiki-100	5.54 b	1.61 a	57.79 b	279.20 a
Koroneiki-200	4.47 b	1.30 b	45.85 b	295.43 a
Arbequina-0	7.96 a	1.83 a	108.32 a	329.36 b
Arbequina-100	4.60 b	1.85 a	105.90 a	394.26 a
Arbequina-200	4.79 b	1.86 a	53.47 b	368.24 ab
**240 DAT**				
Fadak 86-0	5.01 a	2.11 a	72.66 a	266.25 b
Fadak 86–100	-0.68 b	1.28 b	37.53 b	341.68 a
Royal-0	7.77 a	2.34 a	86.55 a	265.92 b
Royal-100	1.78 b	1.55 b	50.04 b	371.09 a
Royal-200	-0.45 b	1.37 b	36.37 b	393.99 a
Koroneiki-0	4.06 a	2.96 a	95.40 a	296.85 b
Koroneiki-100	-0.16 b	1.94 b	53.15 b	375.65 a
Koroneiki-200	-0.83 b	2.11 b	59.32 b	395.95 a
Arbequina-0	2.70 a	4.57 a	167.90 a	333.34 b
Arbequina-100	-0.86 b	3.03 a	95.16 b	383.40 a


*Mean values followed by different letters are significantly different (*P* < 0.05). Tests were performed inside each cultivar for three treatments (0, 100, and 200 mM NaCl) and separately for each time point.*

### Relative Water Content (RWC) and Chlorophyll Content

In general in stressed plants, RWC was lower than control starting from 210 DAT without differences between 100 and 200 mM NaCl (Table 2).

**Table 2:** Relative water content of leaves in four olive cultivars treated with 0, 100, and 200 mM NaCl.

	RWC (%)		
	
	180 DAT	210 DAT	240 DAT
Fadak 86-0	84.36 a	84.36 a	70.40 a
Fadak 86–100	84.15 a	84.15 a	56.65 b
Fadak 86–200	83.37 a	83.37 a	0
Royal-0	82.72 a	74.89 a	76.06 a
Royal-100	83.43 a	76.15 a	66.23 b
Royal-200	79.05 a	77.47 a	64.58 b
Koroneiki-0	88.43 a	77.84 a	75.13 a
Koroneiki-100	84.04 a	81.46 a	72.01 a
Koroneiki-200	83.49 a	78.33 a	64.67 b
Arbequina-0	86.03 a	85.53 a	78.75 a
Arbequina-100	82.73 b	77.44 b	68.51 b
Arbequina-200	80.09 b	73.95 b	0


*Mean values followed by different letters are significantly different (*P* < 0.05). Tests were performed inside each cultivar for three treatments (0, 100, and 200 mM NaCl) and separately for each time point.*

In all plants under saline stress of the four cultivars, low values of chlorophyll content than control were observed at 240 DAT, however, the major impact was observed in stressed plants of “Fadak 86” (Table 3).

**Table 3:** Chlorophyll content of leaves of different olive cultivars treated with 0, 100, and 200 mM of NaCl.

	SPAD		
	
	180 DAT	210 DAT	240 DAT
Fadak 86-0	85.75 a	88.02 a	82.10 a
Fadak 86–100	85.70 a	80.78 b	65.23 b
Fadak 86–200	84.48 a	79.84 b	0
Royal-0	90.74 a	91.29 a	90.40 a
Royal-100	93.08 a	83.22 b	68.00 b
Royal-200	92.14 a	84.39 b	66.99 b
Koroneiki-0	92.99 a	93.18 a	89.53 a
Koroneiki-100	90.54 a	80.96 b	77.18 b
Koroneiki-200	93.44 a	82.33 b	62.51 b
Arbequina-0	95.94 a	93.11 a	94.35 a
Arbequina-100	95.36 a	82.86 b	50.99 b
Arbequina-200	96.78 a	85.62 ab	0


*Mean values followed by different letters are significantly different (*P* < 0.05). Tests were performed inside each cultivar for three treatments (0, 100, and 200 mM NaCl) and separately for each time point.*

### Plant Growth

Salt treatments reduced dry weight (DW) in all plant parts of all examined cultivars at 100 and 200 mM NaCl. The larger DW reductions were observed in leaves while roots and shoots DW decreased only at 200 mM (Table 4 and Supplementary Figures S1–S4). The major DW reduction was observed in “Fadak 86.” Moreover, “Fadak 86” and “Arbequina” showed the lowest RGR values (data not shown).

**Table 4:** Dry weight (DW) (g) of different parts of olive plants treated with 100 and 200 mM NaCl at 240 DAT.

	Roots DW	Shoots DW	Leaves DW	Stem DW	Plant DW
Fadak 86-0	28.81 a	2.63 a	8.19 a	9.85 a	49.48 a
Fadak 86–100	19.06 b	2.66 a	8.36 a	10.56 a	40.64 a
Fadak 86–200	9.52 c	2.51 a	0.03 b	11.10 a	23.16 b
Royal-0	32.24 a	9.30 a	23.14 a	19.11 a	83.79 a
Royal-100	17.40 b	5.20 b	10.19 b	14.99 b	47.78 b
Royal-200	18.52 c	3.84 c	2.78 c	12.11 c	37.25 b
Koroneiki-0	33.54 a	11.87 a	21.75 a	21.64 a	88.80 a
Koroneiki-100	11.38 b	3.83 b	5.81 b	11.26 b	32.28 b
Koroneiki-200	15.61 b	5.03 c	3.21 b	12.30 b	36.15 b
Arbequina-0	25.75 a	9.81 a	24.41 a	21.70 a	81.67 a
Arbequina-100	20.24 b	4.42 b	8.98 b	15.25 b	30.89 b
Arbequina-200	12.35 c	2.16 c	0.02 c	11.99 c	26.52 b


*Mean values followed by different letters are significantly different (*P* < 0.05). Tests were performed inside each cultivar for three treatments (0, 100, and 200 mM NaCl).*

### Enzymatic Activity

**FIGURE 1:**
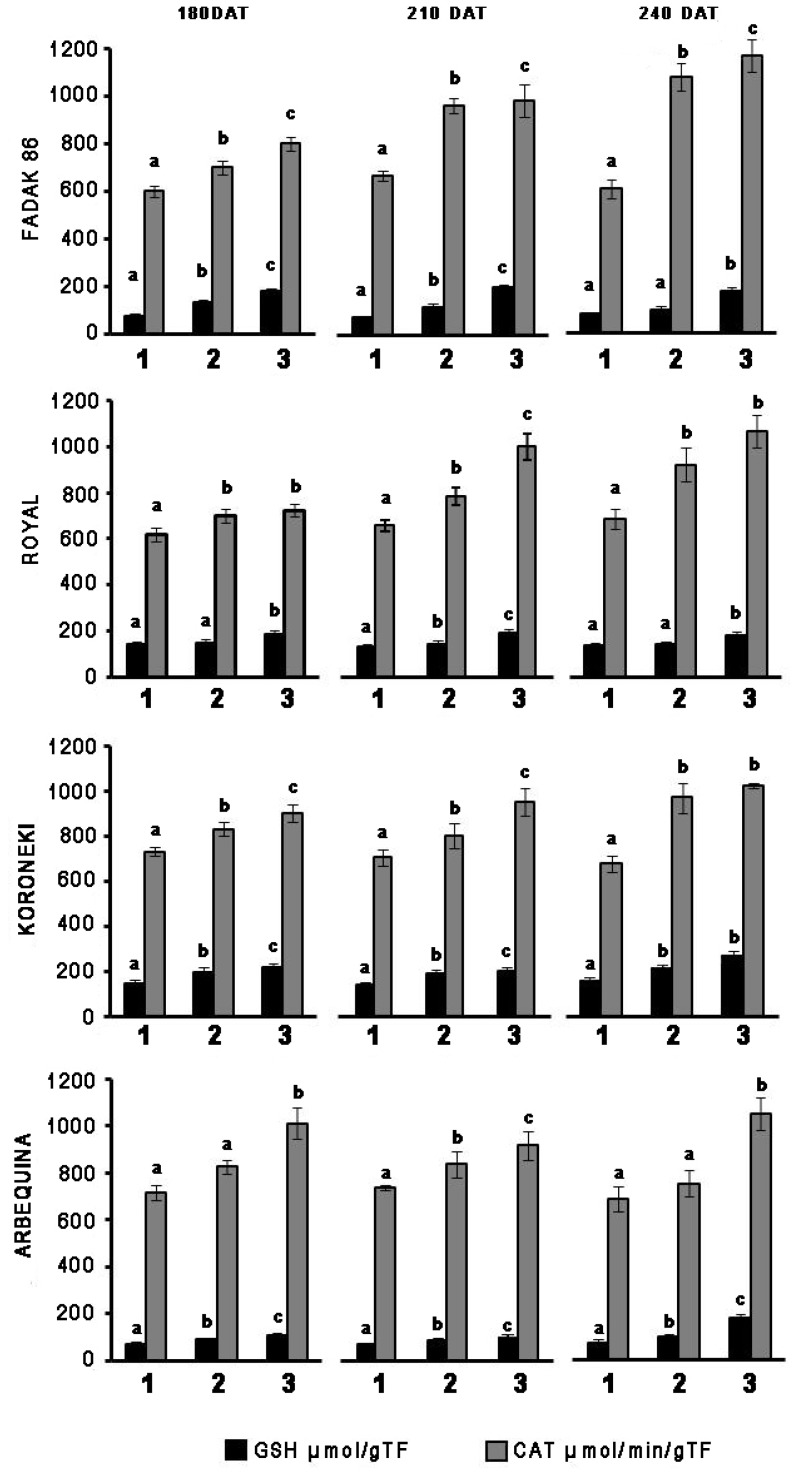
Time-points of glutathione reductase (GSH) and catalase (CAT) activity in leaves of four olive cultivars under saline stress. (1) control, (2) 100 mM NaCl, and (3) 200 mM NaCl. Each value represents the average of five experiments ± SEM. Mean values followed by different letters are significantly different (*P* < 0.05).

In leaves of plants treated with 100 and 200 mM NaCl, GSH, and CAT activities systematically increased in relation to controls across the four cultivars and regardless of the duration of NaCl treatment (Figure 1). CAT increased more markedly with extended exposure to saline stress in “Fadak 86,” “Royal” and, less evidently, in “Koroneiki” and ‘”Arbequina,” whereas GSH activity exhibited higher values in “Royal” plants.

Catalase increased more markedly with prolonged exposure to salt stress in “Fadak 86,” “Arbequina,” and “Koroneiki,” less noticeably, in “Royal,” while GSH activity showed higher values in plants “Koroneiki.”

At 240 DAT the enzymatic activity was determined in the leaves of “Arbequina,” and “Fadak 86” of the few survived trees treated with 200 mM NaCl.

### Proline

**FIGURE 2:**
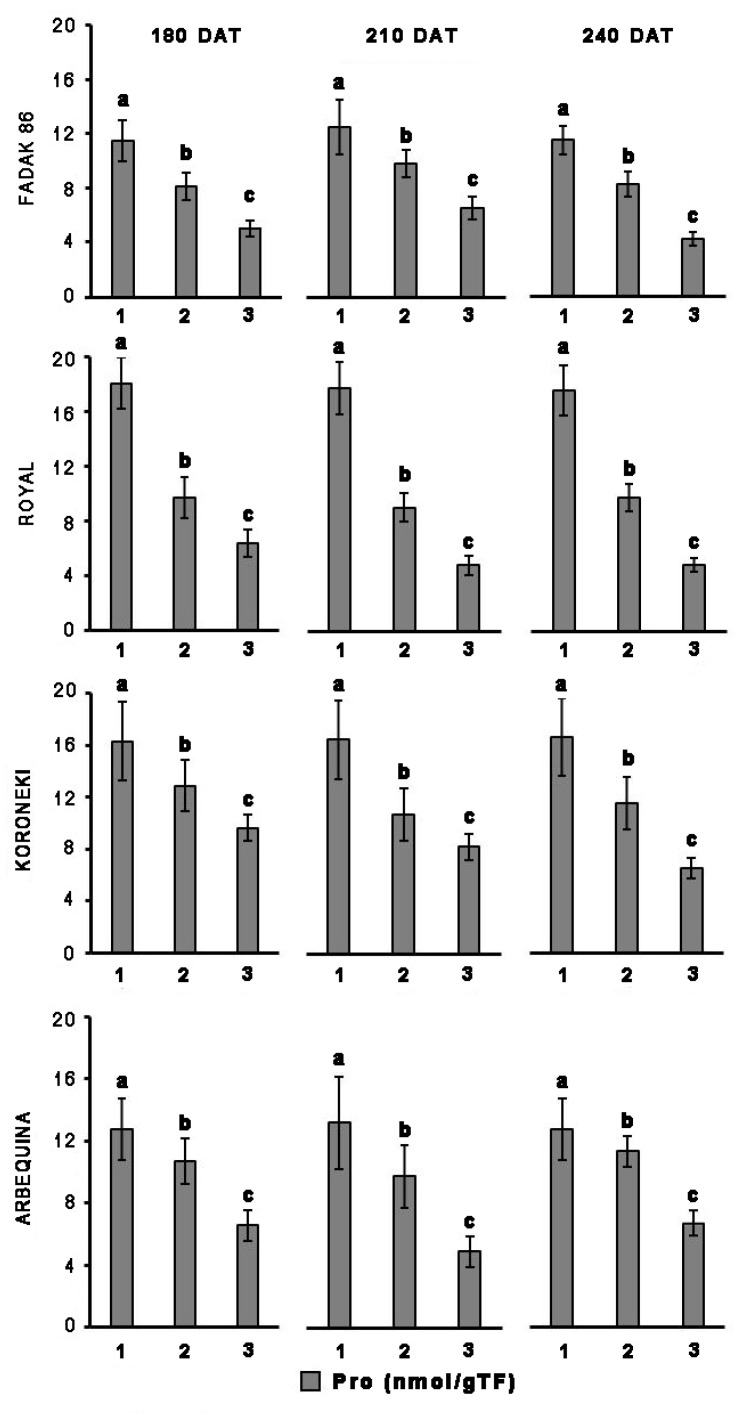
Proline levels in leaves of four olive cultivars under saline stress. (1) control, (2) 100 mM NaCl, and (3) 200 mM NaCl. Each value represents the average of five experiments ± SEM. Mean values followed by different letters are significantly different (*P* < 0.05).

Upon salt stress, proline concentration decreased in all four cultivars, and more evidently at the higher dose of NaCl (Figure 2). Notably, proline reduction did not correlate with duration of the induced stress. In control plants, the concentration of proline in leaves was higher in “Royal” and “Koroneiki” compared to “Arbequina” and “Fadak 86.” At 240 DAT the proline concentration was determined in the leaves of “Arbequina,” and “Fadak 86” of the few survived trees treated with 200 mM NaCl.

## Discussion

In all the four cultivars considered in this study, Pn reduction in leaves under stress conditions was associated with an increase in Ci. This is in agreement with Chartzoulakis (2005) who reports that low and moderate salinity is associated with reduction of CO_2_ assimilation rate. The increase in Ci due to reduction of Pn caused stomatal closure, with a consequent decrease in gs and E and this is in agreement with what postulated by Proietti et al., 2013. The Ci increase is likely indicative that Pn reduction is mainly caused by non-stomatal effects, and could be the result of a damage in the photosystem under saline stress (Proietti and Famiani, 2002; Ben Ahmed et al., 2010; Singh and Reddy, 2011; Proietti et al., 2012).

The reduction of the photosynthetic rate in plants exposed to salt stress togheter with the reduction in Leaf area caused a reduction of plant’s growth (Chartzoulakis et al., 2002; Karimi and Hasanpour, 2014; Pandolfi et al., 2017; Abdallah et al., 2018). In this regard, we noted that saline stressed plants clearly displayed, with time, a lower DW than controls. The DW reduction, mainly localized in leaves was observed also by Karimi and Hasanpour (2014), who found that if the amount of salt rises to a toxic level in the leaves, it causes premature leaf senescence and abscission.

Catalase and GSH enzymes were both investigated in light of their different cellular localization, since CAT is expressed in peroxisomes and removes H_2_O_2_ produced by the conversion of superoxide anion (Guerfel et al., 2009; Huang et al., 2012), while GSH is mainly present in chloroplasts and mitochondria, where it maintains a high ratio between reduced (GSH) and oxidized (GSSG) glutathione, despite formation of GSSG as a result of exposure to the superoxide anion (Bray, 2000; Yousuf et al., 2012; Keunen et al., 2013).

In the absence of stress, “Fadak 86,” “Koroneiki,” and “Arbequina” plants showed a greater activity of CAT, while only the cultivar “Koroneiki” expressed GSH. This may explain the greater resistance of ’Koroneiki’ to saline stress also confirmed by a lower reduction of Pn compared to “Fadak 86” and “Arbequina.”

Decrease in Pn and chlorophyll content after treatments with 100 and 200 mM NaCl, could be related to a greater catalytic activity of both CAT and GSH in the leaves of the four cultivars (Yasar et al., 2008; Sevengor et al., 2011; Keshavkant et al., 2012).

Overall, obtained results indicated significant increase in CAT and GSH enzymatic activities according to the increase of NaCl concentrations across the four cultivars, excepting for GSH at low salt level (100 mM) in “Royal.” The increased activity of CAT and GSH in response to the reduction of Pn indicates an altered redox state in the different cellular compartments of leaves of the four cultivars and can be considered an important marker of cellular response to saline stress, as also reported by Hernández et al. (2000). Indeed a low chlorophyll content in leaves of stressed plants, as observed in the olive plants, is a typical effect of NaCl exposure, associated with an increase of oxidative stress and, at the same time, an increase in ROS scavenging enzymes as a physiological response (Yasar et al., 2008; Gill and Tuteja, 2010; Saha et al., 2010; Din et al., 2011; Arjenaki et al., 2012).

Other reports on olive response to salt stress have shown that the concentration in leaves and roots of osmolytes such as proline, may refer to a possible mechanism of adaptation to unfavorable conditions (Feller and Vaseva, 2014; Iqbal et al., 2014). Proline promotes water retention in the cytoplasm and its higher content appears to represent a specific mechanism engaged by the plants to better tolerate stress conditions (Parida and Das, 2005; Ben Ahmed et al., 2010; Hayat et al., 2012; Iqbal et al., 2014). However, the involvement of osmolytes in carrying out a protective action under unfavorable environmental conditions is currently widely debated and has not yet been elucidated and clarified, considering that tolerance to dehydration also depends on the ability of cells to keep membranes intact and prevent protein denaturation (Munns and Tester, 2008; Iqbal et al., 2014; Cardi et al., 2015). Proline concentration in the four cultivars examined under non-stress conditions was different, that is, higher in “Royal” and “Koroneiki” and less pronounced in “Fadak 86” and “Arbequina.” In saline stressed plants, proline decrease was statistically significant across the four cultivars. This finding is in agreement with the observations of some authors (Ayala-Astorga and Alcaraz-Meléndez, 2010), however, it does not parallel what is reported by others, since in some cases it was shown an increase in proline during saline stress (Ben Ahmed et al., 2009). In this regard, it must be noted that the trend affecting proline concentration is likely associated with NaCl doses higher than 100 mM, usually used to evoke a saline stress (Ayala-Astorga and Alcaraz-Meléndez, 2010). Furthermore, a number of observations concerning proline and saline stress were made under different experimental conditions (e.g., NaCl doses), and by the application of different assay methods, such as non-specific, colorimetric determinations (e.g., ninhydrin), that might impact specificity and sensitivity of the determinations (Bates et al., 1973).

In conclusion, the increase of CAT and GSH in salt stress, induced by high levels of NaCl on the cultivars examined, indicates the presence of a high oxidative stress in progress.

In particular, the Koroneiki cultivar showed a greater response to saline stress, probably due to the prevalence of CAT and GSH in control coditions.

Therefore, it will be interesting to investigate whether the increased activity of CAT, GSH and proline, in basal conditions, may represent a possible prognostic marker of olive trees in the salt stress response.

## Author Contributions

PP, LB, CP, LR, and SM: conceptualization. PP, LR, SM, RD’A, ADP, RM, and HM: investigation. LR, SM, ADP, PP, LB, and CP: methodology and writing original draft. PP, LB, CP, and TG: supervision.

## Conflict of Interest Statement

The authors declare that the research was conducted in the absence of any commercial or financial relationships that could be construed as a potential conflict of interest.

## Supplementary Material

FIGURE S1 Arbequina trees at 240 DAT.

FIGURE S2 Fadak86 trees at 240 DAT.

FIGURE S3 Koroneiki trees at 240 DAT.

FIGURE S4 Royal trees at 240 DAT.

TABLE S1 Gas exchanges – variable Pn.

TABLE S2 Gas exchanges – variable E.

TABLE S3 Gas exchanges – variable gs.

TABLE S4 Gas exchanges – variable Ci.

TABLE S5 Variable RWC.

TABLE S6 Variable chlorophyll content.

TABLE S7 Variable roots DW.

TABLE S8 Variable shoots DW.

TABLE S9 Variable stem DW.

TABLE S10 Variable leaves DW.

TABLE S11 Variable total plant DW.

TABLE S12 Variable GSH.

TABLE S13 Variable CAT.

TABLE S14 Variable proline.
