# Supplementary figures and images for: Behavior of Four Olive Cultivars During Salt Stress
Source: Front Plant Sci. 2019 Jul 5;10:867. doi: 10.3389/fpls.2019.00867 (PMC6624443; doi:10.3389/fpls.2019.00867)

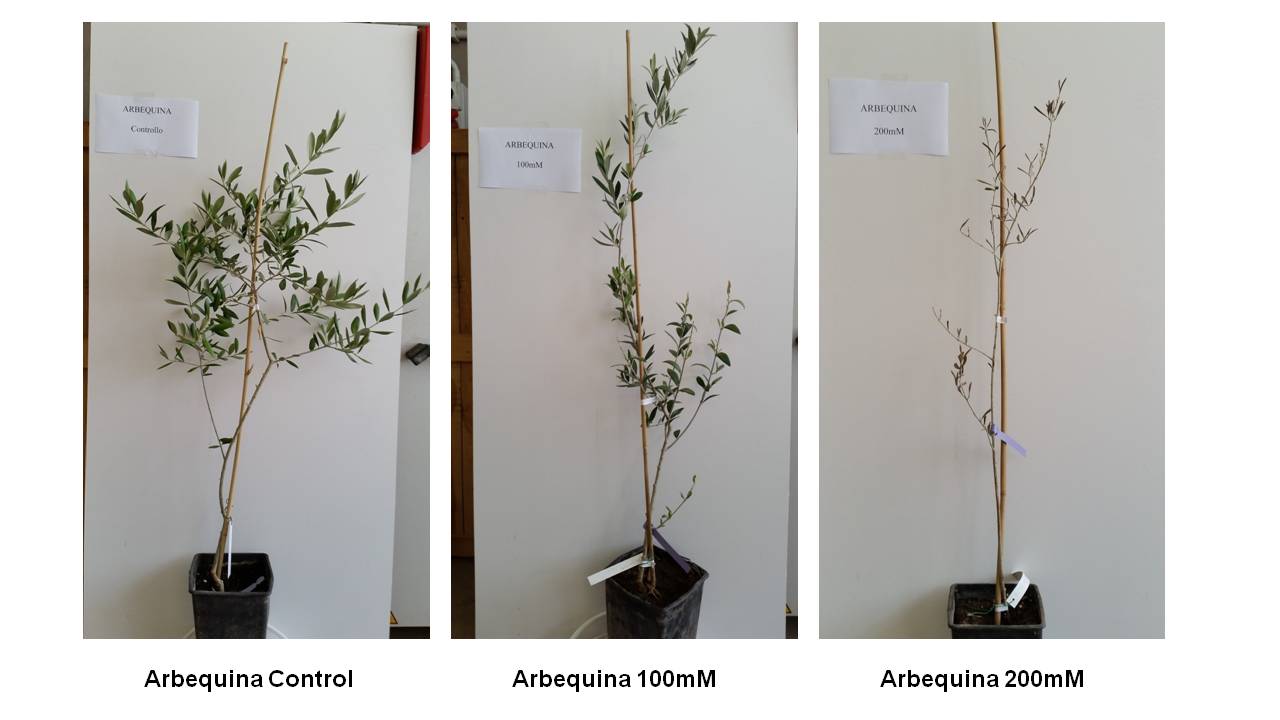

Supplement: FIGURE S1 — Arbequina trees at 240 DAT. [file Image_1.jpg]

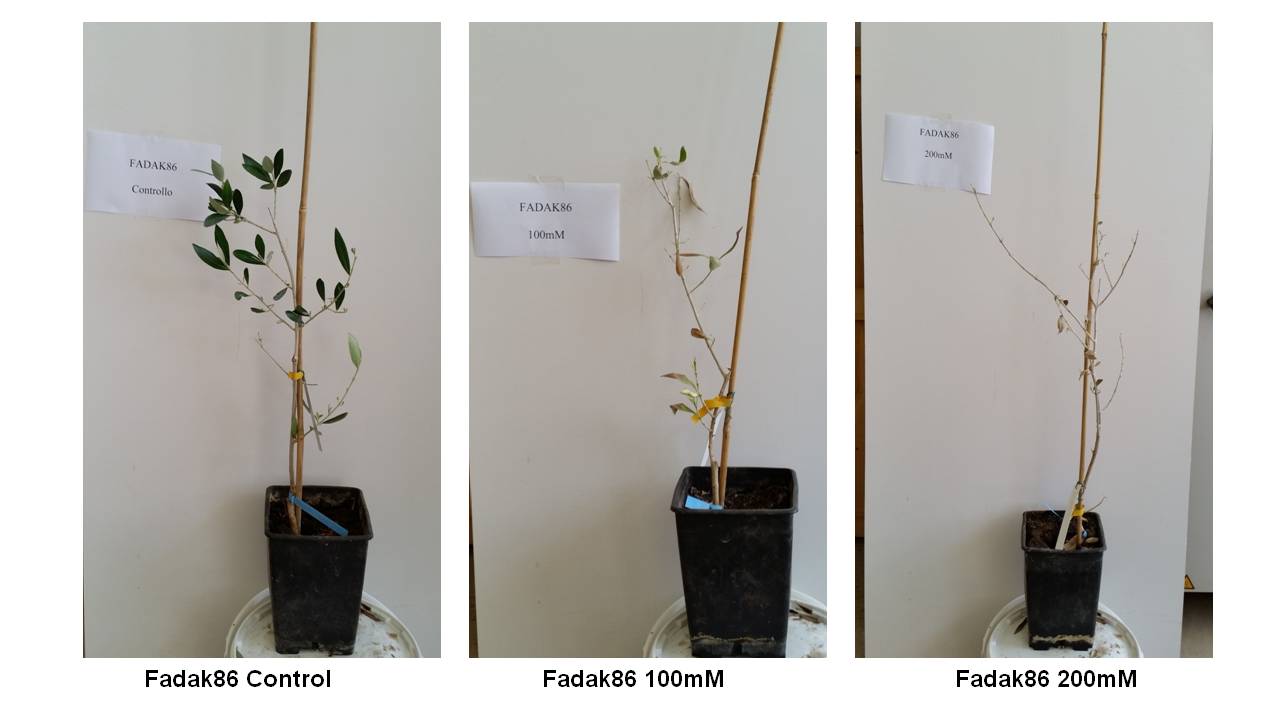

Supplement: FIGURE S2 — Fadak86 trees at 240 DAT. [file Image_2.jpg]

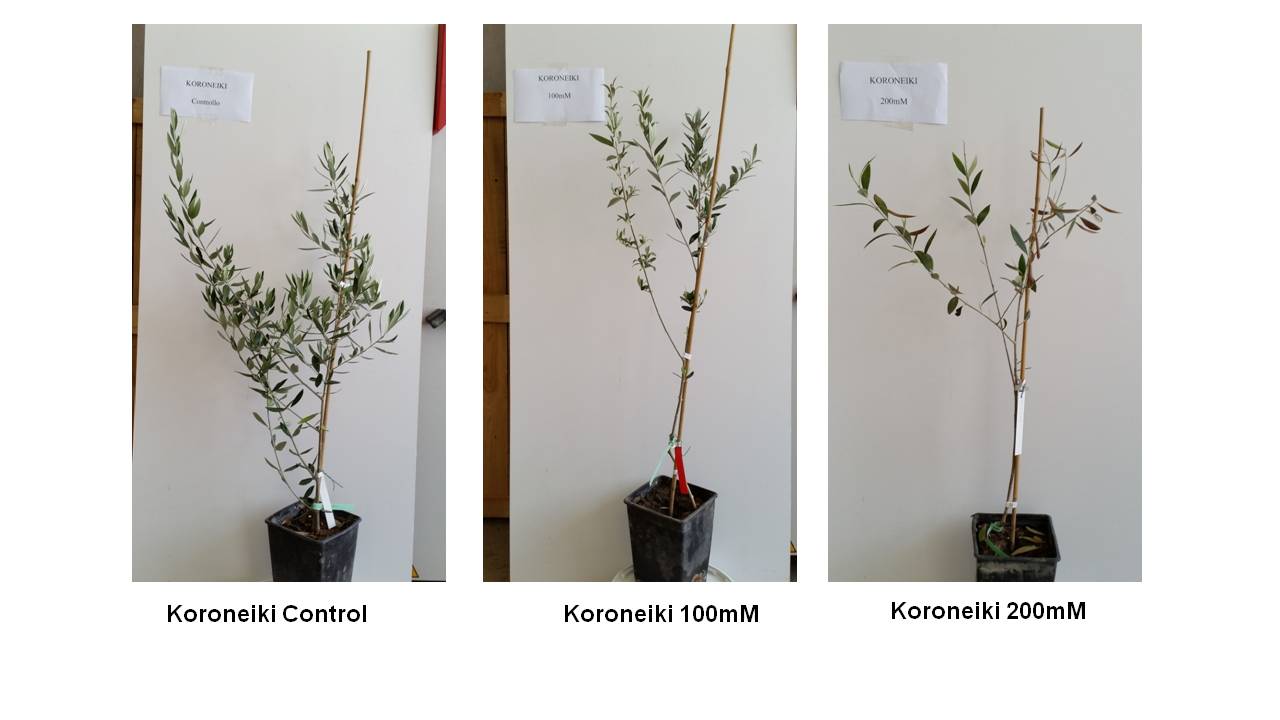

Supplement: FIGURE S3 — Koroneiki trees at 240 DAT. [file Image_3.jpg]

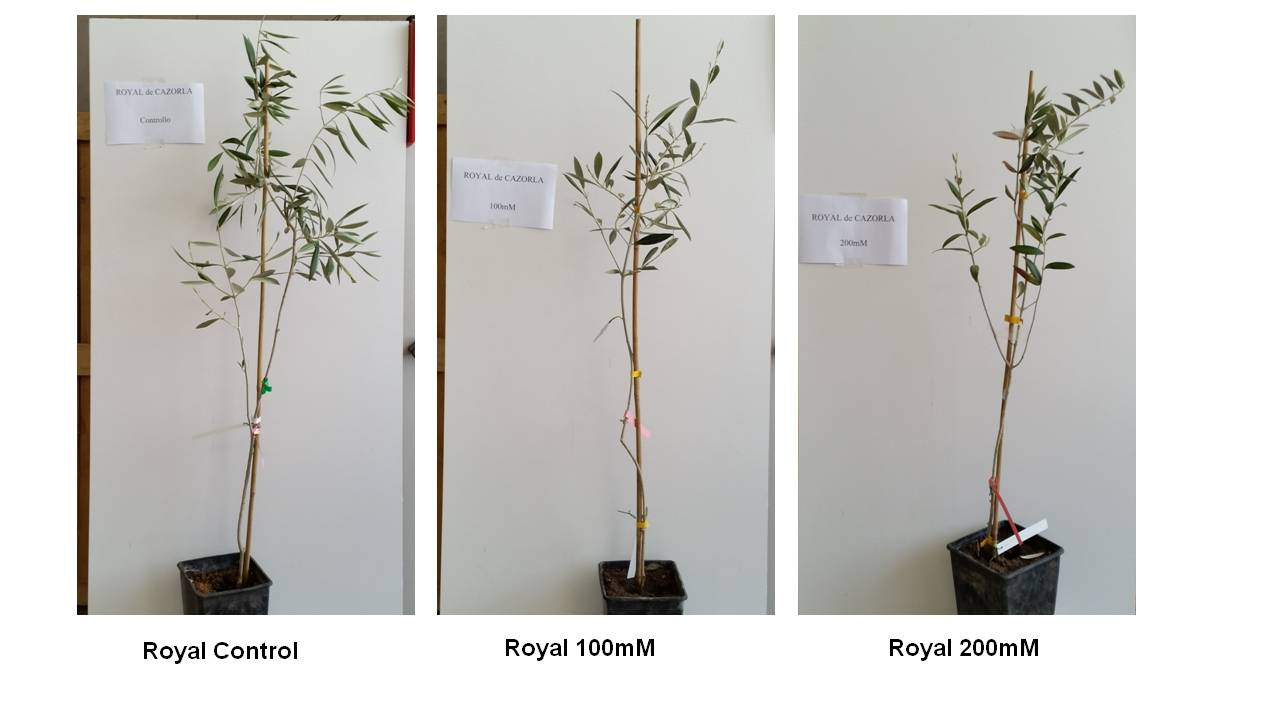

Supplement: FIGURE S4 — Royal trees at 240 DAT. [file Image_4.jpg]
