# Supplementary material for: Behavior of Four Olive Cultivars During Salt Stress
Source: Front Plant Sci. 2019 Jul 5;10:867. doi: 10.3389/fpls.2019.00867 (PMC6624443; doi:10.3389/fpls.2019.00867)
Supplement: TABLE S1 — Gas exchanges – variable Pn. [file Table_1.docx]

Supplementary Table S1. Gas exchanges - variable Pn

| **FACTOR** | **DEVIANCE** | **DF** | **VARIANCE** | **F** | **ProbF** | **Signif.** |
| --- | --- | --- | --- | --- | --- | --- |
| CULTIVAR | 14.42757 | 3 | 4.80919 | 2.04792 | 5.660E-02 | ns |
| SALT CONCENTRATION | 189.2236 | 2 | 94.61181 | 40.28894 | 2.342E-18 | ** |
| TIME | 480.3196 | 2 | 240.1598 | 102.2682 | 5.467E-41 | ** |
| CULTIVARxSALT CONCENTRATION | 9.0667 | 6 | 1.51112 | 0.64348 | 1.399E-01 | ** |
| CULTIVARxTIME | 22.46118 | 6 | 3.74353 | 1.59412 | 1.903E-02 | * |
| SALT CONCENTRATIONxTIME | 29.905 | 4 | 7.47616 | 3.18361 | 8.505E-03 | ** |
| CULTIVARxSALT CONCENTRATIONxTIME | 38.305 | 12 | 3.19209 | 1.3593 | 9.593E-02 | ** |
| Error | 154.9899 | 66 | 2.34833 |  |  |  |
| Total | 2240.598 | 101 | 20.94017 |  |  |  |

*p<0.05 ** p<0.01

Supplementary Table S2. Gas exchanges - variable E

| **FACTOR** | **DEVIANCE** | **DF** | **VARIANCE** | **F** | **ProbF** | **Signif.** |
| --- | --- | --- | --- | --- | --- | --- |
| CULTIVAR | 0.94277 | 3 | 0.31426 | 0.86046 | 1.059E-01 | ns |
| SALT CONCENTRATION | 6.04153 | 2 | 3.02076 | 8.27111 | 6.232E-04 | ** |
| TIME | 49.64244 | 2 | 24.82122 | 67.96265 | 1.110E-16 | ** |
| CULTIVARxSALT CONCENTRATION | 0.89101 | 6 | 0.1485 | 0.40661 | 2.067E-02 | * |
| CULTIVARxTIME | 15.32705 | 6 | 2.55451 | 6.99447 | 8.714E-06 | ** |
| SALT CONCENTRATIONxTIME | 2.023 | 4 | 0.50586 | 1.38509 | 1.101E-02 | * |
| CULTIVARxSALT CONCENTRATIONxTIME | 2.357 | 12 | 0.19641 | 0.5378 | 1.021E-03 | ** |
| Error | 24.10442 | 66 | 0.36522 |  |  |  |
| Total | 122.5167 | 101 | 1.145016 |  |  |  |

*p<0.05 ** p<0.01

Supplementary Table S3. Gas exchanges - variable gs

| **FACTOR** | **DEVIANCE** | **DF** | **VARIANCE** | **F** | **ProbF** | **Signif.** |
| --- | --- | --- | --- | --- | --- | --- |
| CULTIVAR | 3728.23398 | 3 | 1242.745 | 0.77751 | 2.140E-02 | * |
| SALT CONCENTRATION | 18995.07107 | 2 | 9497.536 | 5.94204 | 4.240E-03 | ** |
| TIME | 265847.5671 | 2 | 132923.8 | 83.16243 | 1.892E-24 | ** |
| CULTIVARxSALT CONCENTRATION | 1002.81304 | 6 | 167.1355 | 0.10457 | 4.958E-02 | * |
| CULTIVARxTIME | 40039.44109 | 6 | 6673.24 | 4.17505 | 5.604E-07 | ** |
| SALT CONCENTRATIONxTIME | 2878.574 | 4 | 719.6435 | 0.45024 | 2.296E-02 | * |
| CULTIVARxSALT CONCENTRATIONxTIME | 16088.821 | 12 | 1340.735 | 0.83882 | 1.775E-03 | * |
| Error | 105491.9822 | 66 | 1598.363 |  |  |  |
| Total | 599893.1971 | 101 | 5606.478 |  |  |  |

*p<0.05 ** p<0.01

Supplementary Table S4. Gas exchanges - variable Ci

| **FACTOR** | **DEVIANCE** | **DF** | **VARIANCE** | **F** | **ProbF** | **Signif.** |
| --- | --- | --- | --- | --- | --- | --- |
| CULTIVAR | 3277.7626 | 3 | 1092.588 | 0.50925 | 0.022063 | * |
| SALT CONCENTRATION | 30313.644 | 2 | 15156.82 | 7.06454 | 0.001660 | ** |
| TIME | 144.9174 | 2 | 72.4587 | 0.03377 | 0.049021 | * |
| CULTIVARxSALT CONCENTRATION | 1071.5818 | 6 | 178.597 | 0.08324 | 6.47E-09 | ** |
| CULTIVARxTIME | 267900.79 | 6 | 44650.13 | 20.81126 | 1.52E-22 | ** |
| SALT CONCENTRATIONxTIME | 14490.106 | 4 | 3622.526 | 1.68845 | 0.03568 | ** |
| CULTIVARxSALT CONCENTRATIONxTIME | 18933.387 | 12 | 1577.782 | 0.7354 | 4.24E-08 | ** |
| Error | 141601.66 | 66 | 2145.48 |  |  |  |
| Total | 755266.8 | 101 | 7058.568 |  |  |  |

*p<0.05 ** p<0.01

Supplementary Table S5. Variable RWC

| **FACTOR** | **DEVIANCE** | **DF** | **VARIANCE** | **F** | **ProbF** | **Signif.** |
| --- | --- | --- | --- | --- | --- | --- |
| CULTIVAR | 0.01031 | 3 | 0.00344 | 0.83829 | 4.777E-01 | ns |
| SALT CONCENTRATION | 0.02823 | 2 | 0.01411 | 3.44383 | 1.460E-17 | ** |
| TIME | 0.02764 | 2 | 0.01382 | 3.37164 | 4.680E-29 | ** |
| CULTIVARxSALT CONCENTRATION | 0.00969 | 6 | 0.00162 | 0.39411 | 4.101E-10 | ** |
| CULTIVARxTIME | 0.01117 | 6 | 0.00186 | 0.45417 | 1.986E-13 | ** |
| SALT CONCENTRATIONxTIME | 0.026 | 4 | 0.00644 | 1.57103 | 2.387E-19 | ** |
| CULTIVARxSALT CONCENTRATIONxTIME | 0.019 | 12 | 0.00154 | 0.3769 | 7.977E-12 | ** |
| Error | 0.270502 | 66 | 0.003757 |  |  |  |
| Total | 0.804969 | 101 | 0.007523 |  |  |  |

*p<0.05 ** p<0.01

Supplementary Table S6. Variable chlorophyll content

| **FACTOR** | **DEVIANCE** | **DF** | **VARIANCE** | **F** | **ProbF** | **Signif.** |
| --- | --- | --- | --- | --- | --- | --- |
| CULTIVAR | 1.85803 | 3 | 0.61934 | 0.01662 | 1.709E-03 | ** |
| SALT CONCENTRATION | 1459.65 | 2 | 729.8252 | 19.58843 | 2.097E-07 | ** |
| TIME | 655.8235 | 2 | 327.9117 | 8.80112 | 4.090E-04 | ** |
| CULTIVARxSALT CONCENTRATION | 79.17628 | 6 | 13.19605 | 0.35418 | 4.991E-05 | ** |
| CULTIVARxTIME | 107.1739 | 6 | 17.86231 | 0.47942 | 8.213E-04 | ** |
| SALT CONCENTRATIONxTIME | 964.240 | 4 | 241.06 | 6.47003 | 1.868E-04 | ** |
| CULTIVARxSALT CONCENTRATIONxTIME | 251.566 | 12 | 20.96383 | 0.56267 | 5.174E-23 | ** |
| Error | 2459.026 | 66 | 37.25797 |  |  |  |
| Total | 8632.089 | 101 | 80.67373 |  |  |  |

*p<0.05 ** p<0.01

Supplementary Table S7. Variable roots DW

| **FACTOR** | **DEVIANCE** | **DF** | **VARIANCE** | **F** | **ProbF** | **Signif.** |
| --- | --- | --- | --- | --- | --- | --- |
| CULTIVAR | 71.51666 | 3 | 23.83889 | 0.267736 | 0.847985 | ns |
| SALT CONCENTRATION | 1754.167 | 2 | 877.0837 | 9.85058 | 0.000753 | ** |
| CULTIVAR x SALT CONCENTRATION | 316.3565 | 6 | 52.72609 | 0.59217 | 0.04334 | * |
| Error | 2136.931 | 24 | 89.03879 |  |  |  |
| Total | 4278.972 | 35 | 122.2563 |  |  |  |

*p<0.05 ** p<0.01

Supplementary Table S8. Variable shoots DW

| **FACTOR** | **DEVIANCE** | **DF** | **VARIANCE** | **F** | **ProbF** | **Signif.** |
| --- | --- | --- | --- | --- | --- | --- |
| CULTIVAR | 95.20294 | 3 | 31.73431 | 12.05084 | 5.21E-05 | ** |
| SALT CONCENTRATION | 178.7763 | 2 | 89.38813 | 33.9444 | 1.01E-07 | ** |
| CULTIVAR x SALT CONCENTRATION | 75.14562 | 6 | 12.52427 | 4.755987 | 0.002522 | ** |
| Error | 63.20087 | 24 | 2.633369 |  |  |  |
| Total | 412.3257 | 35 | 11.78073 |  |  |  |

*p<0.05 ** p<0.01

Supplementary Table S9. Variable stem DW

| **FACTOR** | **DEVIANCE** | **DF** | **VARIANCE** | **F** | **ProbF** | **Signif.** |
| --- | --- | --- | --- | --- | --- | --- |
| CULTIVAR | 182.606 | 3 | 60.86865 | 5.683373 | 0.004356 | ** |
| SALT CONCENTRATION | 261.2977 | 2 | 130.6489 | 12.19883 | 0.000221 | ** |
| CULTIVAR x SALT CONCENTRATION | 157.6529 | 6 | 26.27548 | 2.45337 | 0.048138 | * |
| Error | 257.0389 | 24 | 10.70995 |  |  |  |
| Total | 858.5954 | 35 | 24.5313 |  |  |  |

*p<0.05 ** p<0.01

Supplementary Table S10. Variable leaves DW

| **FACTOR** | **DEVIANCE** | **DF** | **VARIANCE** | **F** | **ProbF** | **Signif.** |
| --- | --- | --- | --- | --- | --- | --- |
| CULTIVAR | 227.0562 | 3 | 75.68539 | 6.435739 | 0.002361 | ** |
| SALT CONCENTRATION | 1949.616 | 2 | 974.8082 | 82.89065 | 1.67E-11 | ** |
| CULTIVAR x SALT CONCENTRATION | 340.7394 | 6 | 56.78991 | 4.829004 | 0.00231 | ** |
| Error | 282.2441 | 24 | 11.76017 |  |  |  |
| Total | 2799.656 | 35 | 79.99017 |  |  |  |

*p<0.05 ** p<0.01

Supplementary Table S11. Variable total plant DW

| **FACTOR** | **DEVIANCE** | **DF** | **VARIANCE** | **F** | **ProbF** | **Signif.** |
| --- | --- | --- | --- | --- | --- | --- |
| CULTIVAR | 1801.537 | 3 | 600.5124 | 3.846238 | 0.022208 | * |
| SALT CONCENTRATION | 13196.51 | 2 | 6598.254 | 42.26134 | 1.37E-08 | ** |
| CULTIVAR x SALT CONCENTRATION | 2047.591 | 6 | 341.2652 | 2.185779 | 0.040072 | * |
| Error | 3747.115 | 24 | 156.1298 |  |  |  |
| Total | 20792.75 | 35 | 594.0786 |  |  |  |

*p<0.05 ** p<0.01

Supplementary Table S12. Variable GSH

| **FACTOR** | **DEVIANCE** | **DF** | **VARIANCE** | **F** | **ProbF** | **Signif.** |
| --- | --- | --- | --- | --- | --- | --- |
| Cultivar | 127152 | 3 | 42384.01 | 381.8698 | 4.05E-44 | ** |
| Salt Concentration | 77902.74 | 2 | 38951.37 | 350.9425 | 7.44E-38 | ** |
| Time | 706.463 | 2 | 353.2315 | 3.182531 | 0.047376 | * |
| Cultivar x Salt Concentration | 11196.22 | 6 | 1866.037 | 16.81255 | 5.03E-12 | ** |
| Cultivar x Time | 6694.722 | 6 | 1115.787 | 10.05297 | 4.77E-08 | ** |
| Salt Concentration xTime | 511.9259 | 4 | 127.9815 | 1.153083 | 0.338774 | ns |
| Cultivar x Salt Concentration xTime | 6805.778 | 12 | 567.1481 | 5.109869 | 4.25E-06 | ** |
| Error | 7991.333 | 72 | 110.9907 |  |  |  |
| Total | 238961.2 | 107 | 2233.282 |  |  |  |

*p<0.05 ** p<0.01

Supplementary Table S13. Variable CAT

| **FACTOR** | **DEVIANCE** | **DF** | **VARIANCE** | **F** | **ProbF** | **Signif.** |
| --- | --- | --- | --- | --- | --- | --- |
| Cultivar | 178577.2 | 3 | 59525.74 | 47.42072 | 5E-17 | ** |
| Salt Concentration | 1486866 | 2 | 743433.1 | 592.2503 | 1.97E-45 | ** |
| Time | 450398 | 2 | 225199 | 179.4031 | 1.07E-28 | ** |
| Cultivar x Salt Concentration | 87841.54 | 6 | 14640.26 | 11.66305 | 4.37E-09 | ** |
| Cultivar x Time | 403368.4 | 6 | 67228.07 | 53.55673 | 1.35E-24 | ** |
| Salt Concentration xTime | 175151.5 | 4 | 43787.88 | 34.88328 | 3.5E-16 | ** |
| Cultivar x Salt Concentration xTime | 110083.6 | 12 | 9173.631 | 7.308103 | 1.47E-08 | ** |
| Error | 90379.33 | 72 | 1255.269 |  |  |  |
| Total | 2982666 | 107 | 27875.38 |  |  |  |

*p<0.05 ** p<0.01

Supplementary Table S14. Variable proline

| **FACTOR** | **DEVIANCE** | **DF** | **VARIANCE** | **F** | **ProbF** | **Signif.** |
| --- | --- | --- | --- | --- | --- | --- |
| Cultivar | 179.008 | 3 | 59.66934 | 168.2044 | 1.9E-32 | ** |
| Salt Concentration | 1300.431 | 2 | 650.2157 | 1832.92 | 1.78E-62 | ** |
| Time | 1.733807 | 2 | 0.866904 | 2.443751 | 0.094008 | ns |
| Cultivar x Salt Concentration | 149.9133 | 6 | 24.98555 | 70.43282 | 3.85E-28 | ** |
| Cultivar x Time | 29.34822 | 6 | 4.891369 | 13.78849 | 2.31E-10 | ** |
| Salt Concentration xTime | 4.342525 | 4 | 1.085631 | 3.060332 | 0.021859 | * |
| Cultivar x Salt Concentration xTime | 16.67589 | 12 | 1.389657 | 3.917364 | 0.000125 | ** |
| Error | 25.54149 | 72 | 0.354743 |  |  |  |
| Total | 1706.995 | 107 | 15.95322 |  |  |  |

*p<0.05 ** p<0.01
